# Supplementary material for: Next-generation diabetes diagnosis and personalized diet-activity management: A hybrid ensemble paradigm
Source: PLoS One. 2025 Jan 8;20(1):e0307718. doi: 10.1371/journal.pone.0307718 (PMC11709242; doi:10.1371/journal.pone.0307718)
Supplement: Attachment — Submitted filename: Comments PONE-D-24-07855.docx [file pone.0307718.s002.docx]

1. The motivation for research is not clear from the abstract.
2. The introduction section looks like a thesis writing format. Please change it to a scientific paper format. (Remove the problem statement and contribution heading from the introduction)
3. Please add a full stop at the end of the figure caption.
4. A state of art comparison is missing in the paper.
5. The result analysis should improve further to validate the approach.
6. It is unclear why the authors used Random Forest, Support Vector Machine, and XGBoost models for their study or excluded other ML models.
