# Supplementary material for: Next-generation diabetes diagnosis and personalized diet-activity management: A hybrid ensemble paradigm
Source: PLoS One. 2025 Jan 8;20(1):e0307718. doi: 10.1371/journal.pone.0307718 (PMC11709242; doi:10.1371/journal.pone.0307718)
Supplement: Attachment — Submitted filename: Response to Reviewer ML-DL PLOS.docx [file pone.0307718.s003.docx]

**Reviewer Response to Comments**

1.	The motivation for research is not clear from the abstract.

**Author Response:** Thanks for the comment. We agree with your remarks. The motivation for research has been included in the abstract and it is highlighted.

2.	The introduction section looks like a thesis writing format. Please change it to a scientific paper format. (Remove the problem statement and contribution heading from the introduction)

**Author Response:** Thanks for the comment. We agree with your remarks. The writing format in the introduction section has been changed into a scientific paper format. Problem Statement and Contribution headings both are removed from the introduction. Page 1-3 are highlighted with the required change.

3.	Please add a full stop at the end of the figure caption.

**Author Response:** Thanks for the comment. We agree with your remarks. We have added full stop at the end of each figure caption and highlighted the full stop.

4.	A state of art comparison is missing in the paper.

**Author Response:** Thanks you your valuable comments. A state of art comparison i

5. The result analysis should improve further to validate the approach.

**Author Response:** Thank you for valuable suggestion to improve the result analysis. We have added 2 more Figures 11-12 with different hidden units and learning rate and added some text to describe the figures and further improve the results on pages 14-16. All the changes are highlighted.

6.	It is unclear why the authors used Random Forest, Support Vector Machine, and XGBoost models for their study or excluded other ML models.

**Author Response:** Thanks for the comment. We agree with your remarks. For diabetes prediction, Random Forest, Support Vector Machine (SVM), and XGBoost are often used models. These algorithms were selected because they have a high degree of accuracy in determining if a patient has diabetes or not. These techniques made managing complicated data easier and prevented the model from becoming overfit. In our dataset, these techniques offer versatility in handling various types of data. However, due to low performance necessitating more computation, our approach does not fit with other models such as KNN and Decision Tree. KNN uses a lot of memory and requires additional K values. In contrast, our model's decision tree is unstable and overfit. Not every problem can be solved by every algorithm. Only algorithms that can more accurately predict diabetes and perform better when training and testing the model with the provided dataset are the subject of this study.

7.	We note that Figure 10 in your submission contain copyrighted images.

**Author Response:** Thanks for the comment. We agree with your remarks. Figure 10 is taken from My own Research Paper named RDED which shows workflow for hybrid model. Furthermore this figure has been redrawn and there is no copyright issue in Figure 10.

Few of the Screenshots are given here.

1.	The motivation for research is not clear from the abstract.

2.	The introduction section looks like a thesis writing format. Please change it to a scientific paper format. (Remove the problem statement and contribution heading from the introduction)

3.	Please add a full stop at the end of the figure caption.

4.	A state of art comparison is missing in the paper.

5. The result analysis should improve further to validate the approach.
